# Preface (Part I): quantum theory and topology in models of decision making

**DOI:** 10.1098/rsta.2024.0391

**Published:** 2025-11-27

**Authors:** Jerome R. Busemeyer, Graciela Chichilnisky, Peter Hammond, Emmanuel Haven

**Affiliations:** ^1^ Department of Psychological and Brain Sciences, Indiana University, Bloomington, IN, USA; ^2^ Department of Economics, Columbia University, New York, NY, USA; ^3^ Department of Economics, University of Warwick, Coventry, UK; ^4^ FBA and CQSCS, Memorial University of Newfoundland, St. John's, NL, Canada

**Keywords:** quantum theory, quantum probability, quantum algorithms

An important area of research that continues to thrive explores how the formalism of quantum mechanics has applications that include and transcend physics—for example, it has applications to topology. Past and current research shows how quantum probability can be especially useful in modelling decision making [1–3]. Other notable areas where such research is starting to flourish include economics [4] and biology. Somewhat unrelated to such ‘quantum-like’ applications is the growing area exploring how quantum algorithms can be applied to thorny optimization problems in operations research.

This theme double issue offers a mixture of contributions which highlight recent advances in quantum-like research. These include work that investigates the power, but also the limitations, of quantum algorithms. We also aim to address the key question, not yet answered, of *why* quantum formalism can be useful in the social and behavioural sciences. As Chichilnisky [5, pp. 201−202] explains, in contrast to classical physics, ‘Quantum theory considers all possible experiments on a physical system and breaks tradition by explicitly accepting that there may be no universal experiment and *no single* framework to describe all observed events.’ (Here a ‘framework’ should be read as meaning a basis of coordinates). It is precisely the absence of a single framework which connects us to the paradox of social choice in economics [6,7].

Before briefly delving into each paper in this double issue, we note that some of the work it contains was presented at the workshop on the ‘Applications of Topology to Quantum Theory and Behavioural Economics’. This event was organized by Graciela Chichilnisky and Emmanuel Haven and was held in March 2023 at the Fields Institute for Research in the Mathematical Sciences at the University of Toronto.

The double issue is split into two parts. Part 1 focuses on three core topics:

(i) quantum-like models in psychology;
(ii) quantum-like models in biology and finance;
(iii) philosophical issues related to the foundations of quantum-like research.

Part 2 focuses on the following subjects:

(i) mathematical aspects of quantum probability;
(ii) applications of quantum field theory;
(iii) quantum algorithms.

In this general preface to both parts, we provide some background to each of the papers in the first part, leaving the preface to the second part to provide some details concerning the papers which appear there.

For the first topic, quantum-like models in psychology, Jerome Busemeyer, Masanao Ozawa, Emmanuel Pothos and Naotsugu Tsuchiya focus on the issue of memory in quantum theories of cognition. Then Miho Fuyama, Andrei Khrennikov and Masanao Ozawa discuss the role of non-projective measurements when considering how question order affects survey responses, as well as whether those responses can be replicated. In the subsequent two papers by Diederik Aerts, Massimiliano Sassoli de Bianchi and Sandro Sozzo, ‘concepts’ become quantum entities and the mind is the measuring apparatus. The conceptuality interpretation is laid out in detail in those two articles. Then Johan van der Meer, Pamela Hoyte, Luisa Roeder and Peter Bruza explore how quantum random walk models can describe fluctuations in human trust. William Sulis introduces preference graphs for non-deterministic dynamical systems and investigates intransitivity in human decision making. Ariane Lambert-Moglianski and Irénée Frérot consider contextuality in a quantum cognitive model and focus on contextual opinions. Finally, Raymond Hawkins and Evan Walsh apply a model with contextual probabilities to the projection bias in psychology.

For the second topic, quantum-like models in biology and finance, Fabio Bagarello, Francesco Gargano and Polina Khrennikova apply an open quantum systems approach to an epidemic model that includes both Markovian and semi-Markovian processes. Numerical experiments are also provided. Then Vikram Athalye and Emmanuel Haven introduce a quantum mechanical way of simulating classical stochastic processes and show how this approach can be applied to finance. Next, David Orrell shows how the concept of price impact in finance can be better modelled with quantum mechanics.

For the last topic in this part, philosophical issues related to the foundations of quantum-like research, first Arkady Plotnitsky shows that quantum theory can be seen as a form of decision theory. Two new principles are introduced to finesse the affinities and differences between quantum theory and quantum-like theory. Finally, Terry Robinson illustrates how quantum mechanics can be applied outside physics, based on the argument that von Neumann’s construction of the natural numbers plays a natural role in quantum mechanics.

The papers in the second part of this theme double issue study specific mathematical aspects of quantum mechanics and quantum field theory. That part rounds off the collection with some papers on quantum algorithms.

We hope our theme double issue will stimulate more future research.

## Data Availability

This article has no additional data.
